# A Case of Multisystem Inflammatory Syndrome in a Three-Year-Old Boy

**DOI:** 10.7759/cureus.26315

**Published:** 2022-06-25

**Authors:** Yazeid Alrefaey, Abdulaziz F Alharbi, Yazeed M Alzahrani, Rawia F Albar

**Affiliations:** 1 Medical School, King Saud Bin Abdulaziz University for Health Sciences College of Medicine, Makkah, SAU; 2 College of Medicine, King Saud Bin Abdulaziz University for Health Sciences, Jeddah, SAU; 3 Pediatrics, King Abdulaziz Medical City, Jeddah, SAU

**Keywords:** coronavirus disease, pediatric, sars-cov-2 and covid-19, pediatric case with covid-19, multisystem inflammatory syndrome in children

## Abstract

Multisystem inflammatory syndrome in children (MIS-C) is being recognized in pediatric patients with COVID-19 since mid-2020. Usually, children with MIS-C have systemic symptoms that develop after an infection with SARS-CoV-2, these symptoms can be unremitting fever, gastrointestinal symptoms, skin rashes, conjunctivitis, cardiac or CNS involvement, and shock. We report a case of a three-year-old boy medically free with no prenatal or postnatal abnormalities who presented with three days history of fever and diarrhea. Upon investigation, the patient was found to be COVID-19 polymerase chain reaction (PCR) positive, also had lymphopenia, thrombocytopenia, high inflammatory markers, mildly elevated liver enzymes, high International Normalized Ratio (INR), prothrombin time (PT), partial thromboplastin time (PTT), and upon imaging bilateral peribronchial thickening was noted in a chest X-ray. The patient was treated with intravenous immunoglobulin (IVIG) and other supportive measures were also administered. Eventually, the patient improved, and his inflammatory markers dropped. He was discharged and given a follow-up appointment to further monitor his condition. The findings in this case report correlate with previously published cases that MIS-C have a good prognosis. Although, it is essential that clinicians should be updated on published cases and guidelines to better diagnose, treat, and follow-up MIS-C cases to avoid the long-term sequelae that can affect patients’ lives.

## Introduction

Severe acute respiratory syndrome coronavirus 2 (SARS-CoV-2) is the virus responsible for coronavirus disease 2019 (COVID-19) that began spreading in early 2020. Usually, children with COVID-19 have a self-limiting course, but rarely they can develop multisystem inflammatory syndrome in children (MIS-C) which is a devastating complication of COVID-19 [1]. Usually, the presentation of MIS-C is delayed after SARS-CoV-2 infection, and the clinical features of MIS-C are similar to Kawasaki’s disease in which patients will have an unremitting fever, abdominal pain, vomiting, mucocutaneous lesions, and conjunctivitis. Furthermore, upon laboratory testing and investigations, patients with MIS-C can be found to have multiple abnormalities including but not limited to, lymphopenia, elevated inflammatory markers, cardiovascular involvement in the form of heart failure, pericardial effusion, myocarditis, coronary artery aneurysm, and shock [2-5]. The pathophysiology behind MIS-C is unknown but it is thought to be an immune dysregulation with cytokine release syndrome in response to SARS-CoV-2 infection [5]. The diagnosis of MIS-C can be challenging due to the systemic involvement of the illness, and it requires a high index of suspicion. Therefore, the Center for Disease Control (CDC) has published a case definition for MIS-C [6,7]. The management of MIS-C is still evolving, and it requires a multidisciplinary team. Multiple guidelines have been published but there are no studies that compare the efficacy of treatment options. Management of MIS-C is supportive in the form of fluid replacement, ionotropic, respiratory support, and most patients are given steroids and intravenous immunoglobulin (IVIG), and additional management is based on the case [6,8]. Since May 2020, the CDC has been tracking the cases of MIS-C in the US and they found that the median age of patients with MIS-C is nine years, and White non-Hispanic had the highest percentage of cases compared to other ethnic groups. According to CDC to date, the number of patients that meet the MIS-C case definition in the US is 7459 cases with a total of 63 deaths [9].

The prognosis of MIS-C is yet to be determined due to the novelty of the illness and the lack of long-term follow-up studies. Although it is a serious complication and can be fatal, most cases have survived showing complete resolution [2,10]. The purpose of this report is to describe a case of MIS-C in an especially young patient (three-year-old boy) who was managed and recovered successfully from his illness.

## Case presentation

This is a case of a three-year-old boy known to have an allergy to penicillin who presented to the emergency department with fever and diarrhea for three days. The patient was medically and surgically free (without any previous conditions). He is a full-term and was born with a spontaneous vaginal delivery without any previous pediatric intensive care unit admission. He is the only child of his parents with no family history of hematological, immunological, or genetic disease. There was a history of contact with COVID-19 patient (his parent). COVID-19 swab was taken and confirmed that the patient was COVID-19 positive, therefore he was admitted to the general pediatric ward and isolated.

The patient was in his usual state of health until three days ago when he started to have a fever that reached 38°C which was relieved by an acetaminophen suppository. Fever was associated with diarrhea twice a day that was brown in color, watery in nature, and large in amount (comes out from diaper) without mucus or blood in the stool. Also, he was presented with decreased oral intake consuming only liquids, minimal tears during crying, and reduced activity. Also, urination decreased from three times a day to once a day without changing color and smell. There was no associated history of seizures, photophobia, abdominal pain, vomiting, palpitations, shortness of breath, joint pain or swelling, and urinary complaints.

Upon admission, the patient vital signs were stable (Table 1). On physical examination, he was sleepy and dehydrated but was not in respiratory distress and neither cyanosed nor jaundiced. The patient did not have conjunctivitis, lymphadenopathy, or skin rash. Upon CNS examination, the patient was sleepy but when aroused, he was alert and moving all his limbs and crying. Chest examination showed bilateral equal air entry with no added sound. On cardiovascular examination, first and second heart sounds were loud with no murmur. An examination of the abdomen revealed no abnormalities, it was soft and lax with no tenderness. His initial blood workup and COVID-19 polymerase chain reaction (PCR) were taken.

**Table 1:** Vital signs and body measurement SBP: systolic blood pressure; DBP: diastolic blood pressure; HR: heart rate; RR: respiratory rate

Vital sign	Value
SBP (mmHg)	114
DBP (mmHg)	67
HR (Freq./min)	126
RR (Freq./min)	30
Temperature	37.2
SpO_2_ (%)	97
Body measurement	Value
Height (cm)	97
Weight (kg)	15.5
BMI	16.47

**Figure 1:**
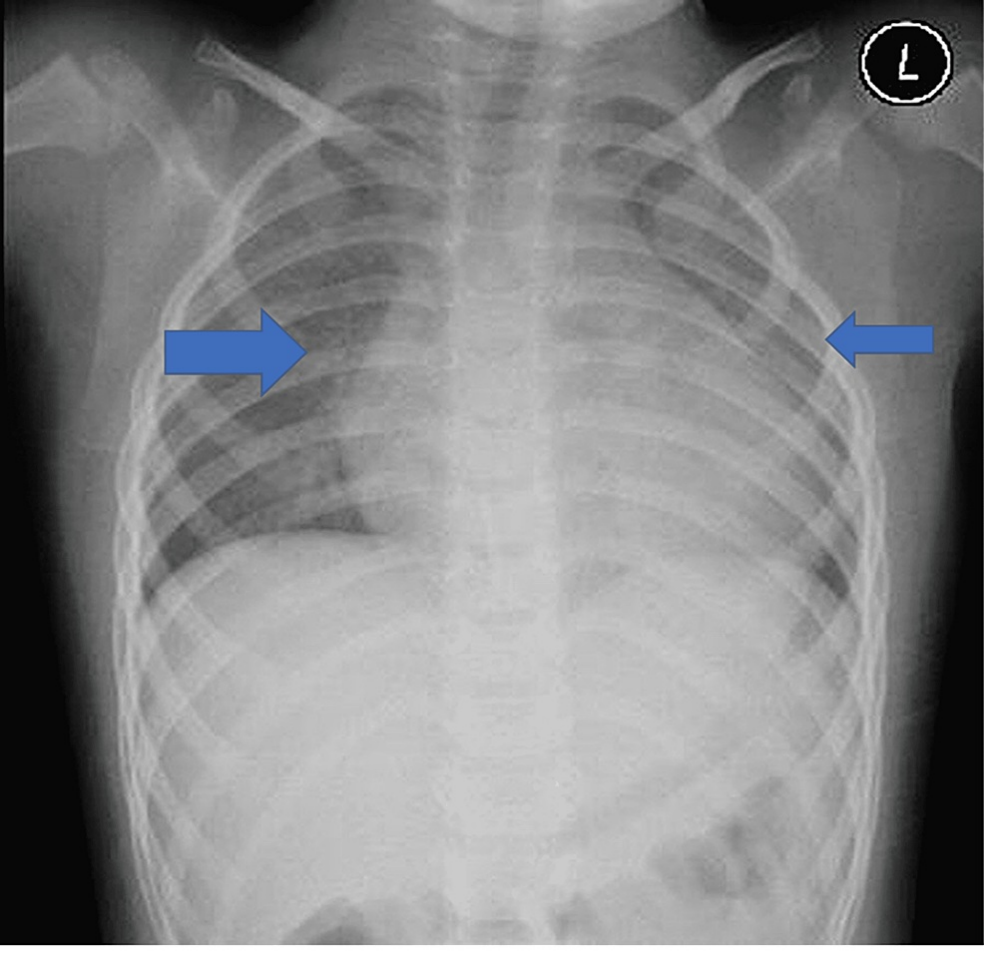
X-ray shows clear lungs fields apart from the bilateral peribronchial thickening (blue arrows).

Results of laboratory tests showed abnormal blood cell counts with leukopenia, lymphopenia, neutropenia, and thrombocytopenia, high inflammatory markers notably C-reactive protein (CRP), procalcitonin (PCT), lactate dehydrogenase (LDH), and ferritin. Also, mildly elevated liver enzymes aspartate aminotransferase (AST), alanine aminotransferase (ALT), and gamma-glutamyl transferase (GGT), mild elevation of D-dimer (DD), high International Normalized Ratio (INR) and prolonged prothrombin time (PT) and partial thromboplastin time (PTT) (Table 2). His COVID-19 PCR report was positive within 24 hours. Routine blood culture was done and the result was negative. An X-ray was done and cardiac size could not be assessed. It also showed clear lung fields apart from the bilateral peribronchial thickening (blue arrows in Figure 1). There were no signs of effusion nor pneumothorax.

**Table 2:** The results of laboratory testing at admission MCHC: mean corpuscular hemoglobin concentration; PCT: pro-calcitonin; CRP: C-reactive protein; LDH: lactate dehydrogenase; ESR: erythrocyte sedimentation rate; INR: International Normalized Ratio; PT: prothrombin time; PTT: partial thromboplastin time; AST: aspartate transaminase; ALT: alanine transaminase; GGT: gamma-glutamyl transferase; BNP: brain natriuretic peptide; CK-MB: creatine kinase myocardial band

Test	Result	Reference value
Blood routine		
Hemoglobin (g/dL)	12.5	11.6-15
Hematocrit (%)	37.4	35.5-44.9
White cell count (*10^9/L)	3.5	3.5-9.5
Platelet count (*10^9/L)	104	125-350
Lymphocyte (*10^9/L)	1.88	2.7-12
Neutrophil (*10^9/L)	0.14	0.6-5.1
Monocyte (*10^9/L)	0.16	0.2-1.4
Eosinophil (*10^9/L)	0.01	0.1-1
MCHC (g/dL)	37	31.5-35
Inflammatory markers		
PCT (µg/L)	1.58	>0.25
CRP (mg/L)	5.4	0-5
LDH (U/L)	336	192-321
Ferritin (µg/L)	1926	5.3-99.9
ESR (mm/h)	2	0-15
Coagulation profile		
D-dimer (mg/L)	1.1	0.17-0.5
INR	1.4	0.8-1.2
PT (s)	16	11-14
PTT (s)	50	26-41
Liver enzymes		
AST (IU/L)	64	21-44
ALT (IU/L)	47	9-25
GGT (IU/L)	54	7-21
Cardiac enzymes		
BNP (pg/mL)	22	10-100
Troponin I (pg/mL)	12.4	≤15.6
CK-MB (ng/mL)	2	0.7-5
Blood culture	Negative	Negative
COVID-19 PCR	Positive	Negative

The day after admission, the patient had a spike of fever of 38.2°C. At that time, the patient was receiving intravenous fluid, acetaminophen, and a single infusion over eight hours of IVIG 2 g/kg. After three days, the fever subsided and there was a good response after administering IVIG in terms of patient activity and inflammatory markers. After 24 hours, the patient was afebrile and hemodynamically stable, he was discharged and scheduled for a follow-up appointment to further monitor his condition and to detect any possible long-term sequelae.

## Discussion

People of various ages, including children, have been impacted by the COVID-19 pandemic. One of the COVID-19 sequelae that can lead to major complications is MIS-C. In children with COVID-19, the pathophysiology of MIS-C is thought to be an infection that triggers macrophage activation followed by helper T-cell activation. This causes a flood of cytokines to be released, as well as the development of antibodies, resulting in immunological dysregulation and a hyperimmune response. Systemic symptoms of inflammation are commonly evident, along with substantial cardiac, neurologic, and/or hematologic dysfunction [4].

MIS-C consists of six main components which are persistent fever, pediatric age group, high laboratory marker of inflammation, signs or symptoms of organ damage, lack of other diagnoses, and clinically confirmed COVID-19 infection or exposure [11]. According to published data, our case met the MIS-C case criteria, presenting with continuous fever >38°C and diarrhea [12-18]. There is also evidence of elevated inflammatory markers, such as CRP, PCT, and ferritin, as well as negative blood culture, to exclude other infectious diseases [14,19,20]. There was also a history of COVID-19 contact (with his parent), which was confirmed by COVID-19 PCR to be positive. Our case presenting symptoms were only fever and diarrhea, which is an atypical presentation of MIS-C. Our patient did not manifest peripheral edema, mucocutaneous changes, symptoms of conjunctivitis, abdominal pain, vomiting, or chest pain after three days of fever, which is contrary to existing MIS-C literature. Clinical features similar to our case have been documented all over the world. Our patient had a three-day fever, which is common in all the available reported cases. Abdominal pain, diarrhea, and vomiting have been recorded in 60-100% of MIS-C patients, and only diarrhea was seen in our case as well [21-23].

In most MIS-C cases, abnormal white blood cell count with primarily neutrophilia 90%, high acute phase reactants CRP 90-100%, erythrocyte sedimentation rate (ESR) 75-80%, and ferritin 55-76% are found in upon laboratory investigation [4]. Similarly, our case presented with elevated inflammatory markers CRP, PCT, LDH, and ferritin. In addition, children with MIS-C had a higher percentage of distinct laboratory abnormalities including lymphopenia, thrombocytopenia, and elevated ferritin levels [24]. Also, our patient’s laboratory test result showed leukopenia, lymphopenia, neutropenia, and thrombocytopenia. Furthermore, there is evidence that raised cardiac markers like troponin are found in 50-90% of MIS-C cases, as well as hypoalbuminemia, mild increases in liver enzymes, and lactate dehydrogenase in 10-60% of MIS-C cases [4]. In our case, laboratory results showed elevation in liver enzymes and LDH as well, but cardiac enzymes were within the normal range. Moreover, 688 MIS-C patients were recognized as having brain natriuretic peptide (BNP) raised in a published systematic study, and it was present in 86% of them, as for our case it was normal [23]. Additionally, one of the complications that are associated with MIS-C is coagulopathy (prolonged PT or PTT, elevated DD), which our case presented with high INR, prolonged PT, PPT, and elevated DD [25]. It was reported that the majority of the children have been primarily in the 5-14 age groups, while in our case it was a three-year-old of an Arab descent [4].

There have been multiple case series of typical and atypical Kawasaki disease presenting in the setting of COVID-19 in addition to MIS-C [26,5]. Our case is distinct in that our patient’s only symptoms were fever and diarrhea at the time of presentation. For a better knowledge of the pathogenesis and management of MIS-C, multinational and multicenter randomized controlled studies are needed.

## Conclusions

MIS-C is a well-recognized complication of COVID-19 disease and clinicians should approach cases of COVID-19 with a high index of suspicion especially if atypical presentation with multisystemic symptoms. Although the mortality rate from MIS-C is not high, the long-term sequelae are worrisome. The published guidelines and cases should help clinicians better pick MIS-C cases and treat them early on to avoid complications. The management of MIS-C is multidisciplinary, and treatment is individualized according to the involved system. In general, corticosteroids and IVIG are shown to be effective in the treatment of MIS-C, and long-term follow-up is of the essence, especially for children who developed complications.
